# Measuring nasal bacterial load and its association with otitis media

**DOI:** 10.1186/1472-6815-6-10

**Published:** 2006-05-10

**Authors:** Heidi Smith-Vaughan, Roy Byun, Mangala Nadkarni, Nicholas A Jacques, Neil Hunter, Stephen Halpin, Peter S Morris, Amanda J Leach

**Affiliations:** 1 Menzies School of Health Research, Darwin, Australia; 2 Institute for Advanced Studies, Charles Darwin University, Darwin, Australia; 3 Institute of Dental Research, Westmead Millennium Institute and Westmead Centre for Oral Health, Sydney, Australia; 4 Northern Territory Clinical School, Flinders University, Adelaide, Australia

## Abstract

**Background:**

Nasal colonisation with otitis media (OM) pathogens, particularly *Streptococcus pneumoniae, Haemophilus influenzae *and *Moraxella catarrhalis*, is a precursor to the onset of OM. Many children experience asymptomatic nasal carriage of these pathogens whereas others will progress to otitis media with effusion (OME) or suppurative OM. We observed a disparity in the prevalence of suppurative OM between Aboriginal children living in remote communities and non-Aboriginal children attending child-care centres; up to 60% and <1%, respectively. This could not be explained by the less dramatic difference in rates of carriage of respiratory bacterial pathogens (80% vs 50%, respectively). In this study, we measured nasal bacterial load to help explain the different propensity for suppurative OM in these two populations.

**Methods:**

Quantitative measures (colony counts and real-time quantitative PCR) of the respiratory pathogens *S. pneumoniae, H. influenzae *and *M. catarrhalis*, and total bacterial load were analysed in nasal swabs from Aboriginal children from remote communities, and non-Aboriginal children attending urban child-care centres.

**Results:**

In both populations nearly all swabs were positive for at least one of these respiratory pathogens. Using either quantification method, positive correlations between bacterial load and ear state (no OM, OME, or suppurative OM) were observed. This relationship held for single and combined bacterial respiratory pathogens, total bacterial load, and the proportion of respiratory pathogens to total bacterial load. Comparison of Aboriginal and non-Aboriginal children, all with a diagnosis of OME, demonstrated significantly higher loads of *S. pneumoniae *and *M. catarrhalis *in the Aboriginal group. The increased bacterial load despite similar clinical condition may predict persistence of middle ear effusions and progression to suppurative OM in the Aboriginal population. Our data also demonstrated the presence of PCR-detectable non-cultivable respiratory pathogens in 36% of nasal swabs. This may have implications for the pathogenesis of OM including persistence of infection despite aggressive therapies.

**Conclusion:**

Nasal bacterial load was significantly higher among Aboriginal children and may explain their increased risk of suppurative OM. It was also positively correlated with ear state. We believe that a reduction in bacterial load in high-risk populations may be required before dramatic reductions in OM can be achieved.

## Background

For Aboriginal children in remote communities of the Northern Territory of Australia, otitis media (OM) commences within the first weeks of life and progresses to tympanic membrane perforation in 30% of children by 6 months of age [1]. A recent cross-sectional study of Northern Territory Aboriginal children aged 6 to 30 months living in 29 remote communities demonstrated a community perforation prevalence of 0–60% [2]. In contrast, whilst children attending urban child-care centres are also at high risk of OM, the prevalence of tympanic membrane perforation is almost 100 fold lower (less than 1%)[3,4].

For Aboriginal children, we have described simultaneous colonisation with multiple species and strains of *Streptococcus pneumoniae*, *Haemophilus influenzae *and *Moraxella catarrhalis*, which may contribute to persistent and progressive ear disease. Prevalence of each respiratory pathogen among young Aboriginal children is approximately 80% [1] compared to approximately 50% for non-Aboriginal attending child-care centres [5]. The risk of simultaneous nasal carriage with *H. influenzae *and *S. pneumoniae *was nearly three-fold higher in Aboriginal children [6], but these differences were not sufficient to explain the substantial variation in prevalence of suppurative disease between the two populations.

We further believe that the high rates of carriage are driven by early age of colonisation with these multiple strains, acquired by infants through high rates of cross-infection in overcrowded living conditions. There is potential for colonisation in the first weeks of life to result in long carriage times, driven by immune suppression [1], inflammatory processes in direct response to bacterial virulence factors[7], reduced cilial function, and thus failure to clear the multiple strains[8,9]. Treatment failure following antibiotic therapy for otitis media is common in Aboriginal children and can only partially be attributed to low compliance and antibiotic resistance.

We explored the possibility that a high nasal bacterial load is an important determinant in the progression of children to suppurative OM. Other studies have reported the relationship between bacterial load and the severity or clinical course of a number of infections including, colitis in the rat model [10], typhoid fever [11], and meningococcal disease [12]. Furthermore, there exists a relationship between bacterial load and markers of airway inflammation for the pathogenesis of bronchiectasis [13]. Inflammatory markers increased at bacterial loads of 10^6 ^to 10^7 ^cfu/ml, and continued to increase progressively with further bacterial load. Theoretically, therefore, an interruption of disease progression may be achieved by reducing bacterial load.

Thus the aim of this study was to measure bacterial load in nasal swabs from two populations known to be at high and low risk of suppurative OM. Measurements were undertaken using three independent methods for bacterial enumeration; semi-quantitative bacterial culture, serial dilution, and real-time quantitative PCR (RTQ-PCR).

## Methods

### Ethical approval

Ethical approval for this study was granted in 2003 by the Human Research Ethics Committee of the Northern Territory Department of Health and Community Services and Menzies School of Health Research.

### Participants and sample collection

This study utilised nasal swabs from previous studies in two populations. Each swab had been stored in 1.0 ml skim milk-tryptone-glucose-glycerin-broth (STGGB) [14] at -70°C for up to six years. Fifty-one nasal swabs were randomly selected from 59 Aboriginal children aged 18 to 36 months living in a remote Northern Territory community. These children were being assessed as part of the follow up of a randomised placebo-controlled trial of long-term antibiotics for prevention of OM in the first year of life. This cohort of Aboriginal children was known to have a high incidence of both respiratory pathogen carriage and suppurative OM. The second cohort consisted of 52 children randomly selected from 235 non-Aboriginal children aged 18 to 36 months attending Darwin child-care centres. Nasal swabs were collected from these children at baseline (prior to randomisation) as part of a hygiene intervention trial. Stored swabs were thawed on ice, mixed, and 200 μL aliquots removed for use in this study. All aliquots were re-labelled with random identification numbers, thus blinding laboratory staff to the population group, clinical and prior microbiological data. Random samples were generated using STATA version 7 (StataCorp LP, College Station, Tx).

### Ear examinations

Ears were examined using video otoscopy and tympanometry. Tympanic membrane videos and tympanograms were reviewed by a second independent observer. For purpose of this analysis, the child was categorised according to their worse ear.

Categories were:

**No OM **– normal (neutral tympanic membrane with normal mobility and type A tympanogram or retracted tympanic membrane with normal mobility and type C tympanogram).

**OME **– OM with effusion (neutral, retracted or mildly bulging tympanic membrane with reduced mobility and type B tympanogram) and without symptoms of acute infection.

**Suppurative OM **– acute OM (moderate to marked bulging of the tympanic membrane with reduced mobility and type B tympanogram), or acute OM with perforation (fresh discharge through a recent perforation of the tympanic membrane), or dry perforation, or chronic suppurative OM (fresh discharge through a persistent perforation of the tympanic membrane).

### Bacterial load estimates

*S. pneumoniae, H. influenzae, M. catarrhalis*, and total bacterial load were determined as follows:

#### i) Semi-quantitative colony counts

The stored swab in STGGB was thawed, a 10 μl aliquot was plated onto chocolate agar (Oxoid, Adelaide, SA) and streaked in quadrants. Bacterial load was categorised as follows: 0, no growth; 1, <20 bacterial colonies; 2, 20–50 bacterial colonies; 3, 50–100 bacterial colonies; 4, confluent growth in the primary zone; 5, confluent growth in the primary zone and colonies in the secondary zone; 6, confluent growth in the secondary zone and colonies in the tertiary zone; and 7, confluent growth in the tertiary zone and colonies in final streak zone. As previous microbiological analysis of these swabs had identified the species present, colonies counted were selected by morphological identification. Any colonies with uncertain identity were fully characterized as described previously [1].

#### ii) Quantitative serial dilution colony counts

Serial 10-fold dilutions of 10 μl aliquots were made using Mueller-Hinton Broth (Oxoid) and lawn streaked onto chocolate agar plates. Colonies were identified as in (i).

#### iii) Real-time quantitative PCR (RTQ-PCR)

Total DNA was extracted from a 50 μl aliquot of the sample. In brief, cells were incubated in 50 mM phosphate buffer pH 6.7 containing 20 mM diethyl pyrocarbonate, 1 mg lysozyme ml^-1^, 1 mg mutanolysin ml^-1 ^and 2 mg Proteinase K ml^-1 ^at 56°C for 40 min and lysed with 1% (w/v) SDS, prior to DNA extraction and purification using QIAmp DNA Mini kit (QIAGEN, Clifton Hill, Vic) according to the manufacturer's protocol. Genomic DNA of the type strains, *S. pneumoniae *ATCC 6305, *H. influenzae *ATCC 10211 and *M. catarrhalis *ATCC 25238, was extracted from overnight cultures using the QIAmp DNA mini kit, as described above, except that diethyl pyrocarbonate was not included. DNA concentrations were determined with the PicoGreen double-stranded DNA quantification kit (Invitrogen-Molecular Probes, Mulgrave, Vic) and Luminescence spectrophotometer model LS 50B (Perkin Elmer, Melbourne, Vic).

Total bacterial loads were determined using pre-optimised concentrations of the universal forward (5'-TCCTACGGGAGGCAGCAGT-3'; 300 nM) and reverse (5'-GGACTACCAGGGTATCTAATCCTGTT-3'; 300 nM) primers and probe (5'- [6-FAM]-CGTATTACCGCGGCTGCTGGCAC- [TAMRA]-3'; 175 nM) for the 16S rRNA gene, as previously described [15]. Purified genomic DNA of *Streptococcus mitis *strain NCTC 1226, in the range of 100 fg to 1 ng, was used to standardise the values. DNA quantities determined by RTQ-PCR were converted to cells (ml of sample)^-1^, in order to represent the number of bacterial cells per nasal swab, by assuming that 1 pg of DNA was equivalent to 447.4 cells, i.e. a genome size of 2 Mb.

Total *S. pneumoniae *loads were determined using pre-optimised concentrations of the forward (5'-TCTTACGCAATCTAGCAGATGAAGC-3'; 100 nM) and reverse (5'-GTTGTTTGGTTGGTTATTCGTGC-3'; 100 nM) primers and probe (5'- [6-FAM]-TTTGCCGAAAACGCTTGATACAGGG- [TAMRA]-3'; 200 nM) for the autolysin gene, *lytA*, as described previously [16], except that the primers were modified for compatible *T*_*m*_s. Purified genomic DNAof *S. pneumoniae *ATCC 6305, in the range of 20 fg to 200 pg, was used to standardise the values. DNA loads were converted to cells (ml of sample)^-1 ^assuming that 1 pg of DNA was equivalent to 447.4 cells, i.e. a genome size of 2 Mb.

Total *H. influenzae *loads were determined using pre-optimised concentrations of the forward (5'-CTGGWGCAATGGCAGAAGTG-3'; 100 nM) and reverse (5'-TCTTTACGCACGGTGTAAGGATG-3'; 200 nM) primers and probe (5'- [6-FAM]-AATATGCCGATGGTGTTGGYCCAGGTT- [TAMRA]-3'; 100 nM) for the outer membrane protein D gene, *glpQ*, which shows limited sequence variation among *H. influenzae *type b and non-encapsulated strains [17]. Purified genomic DNA of *H. influenzae *ATCC 10211, in the range of 20 fg to 200 pg, was used to standardise the values. DNA quantities were converted to cells (ml of sample)^-1 ^assuming that 1 pg of DNA was equivalent to 497.1 cells, i.e. a genome size of 1.8 Mb.

Total *M. catarrhalis *loads were determined using pre-optimised concentrations of the forward (5'- GTGAGTGCCGCTTTTACAACC-3'; 300 nM) and reverse (5'-TGTATCGCCTGCCAAGACAA-3'; 300 nM) primers and probe (5'- [6-FAM]-TGCTTTTGCAGCTGTTAGCCAGCCTAA- [TAMRA]-3'; 200 nM) for the outer membrane protein gene, *copB*, as described previously [18]. Purified genomic DNA of *M. catarrhalis *ATCC 25238, in the range of 20 fg to 200 pg, was used to standardise the values. DNA quantities were converted to cells (ml of sample)^-1 ^assuming that 1 pg of DNA was equivalent to 497.1 cells, i.e. a genome size of 1.8 Mb.

RTQ-PCR was performed with the ABI-PRISM 7700 Sequence Detection System (Applied Biosystems, Scoresby, Vic) using optical grade 96-well plates in a reaction volume of 25 μl using the TaqMan PCR Core Reagent Kit (Applied Biosystems) containing the forward and reverse primers and the fluorogenic probe. The RTQ-PCR conditions were 50°C for 2 min and 95°C for 10 min, followed by 40 cycles of amplification at 95°C for 15 s and 60°C for 1 min. Reactions were performed in triplicate and the mean values calculated. Data analysis was performed using the Sequence Detection Software version 1.6.3 supplied by Applied Biosystems. Samples that were negative by RTQ-PCR for *S. pneumoniae*, *H. influenzae *or *M. catarrhalis *DNA were checked qualitatively by PCR in 25 μl reactions containing 1 × Platinum PCR SuperMix (Invitrogen), 2 μl of the sample DNA and 200 nM each of the specific primers. PCR was performed using the GeneAmp PCR System 9700 (Perkin Elmer) with an initial denaturation step of 95°C for 10 min, followed by 40 cycles of 95°C for 15 s and 60°C for 1 min. A 10 μl aliquot of the PCR reaction was subjected to electrophoresis on a 2% (w/v) agarose gel containing 1 μg ethidium bromide ml^-1 ^and the DNA bands visualized by UV illumination.

### Statistical analysis

The bacterial load dataset was anticipated to contain a large proportion of zero counts and not to be normally distributed. Therefore non-parametric tests were selected for measuring association between variables. The degree of correlation between bacterial load counts estimated using colony counts and RTQ-PCR was computed as the Spearman correlation coefficient. The difference between groups (bacterial load estimates, ear state) was assessed using the Kruskal-Wallis test, Chi^2 ^exact test, or Mann-Whitney U test. A logistic regression model was developed to predict ear state for various levels of bacterial load estimates. The sensitivity and specificity for predicting ear state were determined for various levels of bacterial load. The association between multiple carriage and ethnicity, as well as the association between cultivable bacteria and ear state were assessed using the 2-tailed Fisher's exact test. All statistical analyses were undertaken using STATA version 8.0 (StataCorp LP).

### Study limitations

This study was limited by the available sampling technology that made it necessary to assume that counts per swab represented the burden of bacterial load in the nose; estimation of total nasal bacterial numbers could be subject to misinterpretation if the volume of secretion in the nose varied substantially.

## Results

### Limits of bacterial detection

RTQ-PCR allowed for the detection of between 6.5 × 10^2 ^to 1.8 × 10^8 ^cells (ml of sample)^-1^. Samples negative by RTQ-PCR were also negative by qualitative PCR after 40 cycles. In the case of serial dilution colony counting, the lowest positive estimate was 1 × 10^3 ^cells (ml of sample)^-1^.

### Comparison of colony counts and RTQ-PCR

Comparison of serial dilution colony counts and RTQ-PCR indicated that 70% and 85% of the 103 swabs were positive for *S. pneumoniae*, 59% and 82% for *H. influenzae*, and 83% and 87% for *M. catarrhalis*, respectively. Despite the consistently higher detection by RTQ-PCR, a strong positive correlation was seen between this method and the serial dilution colony counts method. Spearman correlation coefficients for *S. pneumoniae, H. influenzae *and *M. catarrhalis *were 0.77, 0.66 and 0.83, respectively.

### Detection of pathogens by culture and RTQ-PCR

18%, 23%, and 6% of the 103 swabs were culture-negative but RTQ-PCR-positive for *S. pneumoniae, H. influenzae *and *M. catarrhalis*, respectively. A single swab was negative for all three respiratory pathogens, but was positive for other flora.

Four swabs (4%) were RTQ-PCR-negative but culture-positive; a single colony of the RTQ-PCR-negative pathogen was cultured from 3 swabs at the lowest serial dilution. The remaining swab cultured high numbers of the RTQ-PCR-negative bacterium (*H. influenzae*) suggesting the possible presence of an unknown inhibitor of the PCR process in this sample.

### Prevalence and severity of ear disease and diversity of species in nasal swabs

Aboriginal children from remote communities had a much lower prevalence of no OM and a higher prevalence of OME and suppurative OM than non-Aboriginal children; 6% Aboriginal children had bilaterally normal ears, 43% had OME, and 51% had suppurative OM. In contrast, 65% of the non-Aboriginal group had bilaterally normal ears, 31% OME, and only 4% suppurative OM.

Of the children with a suppurative OM diagnosis, the 2 non-Aboriginal children and 8% Aboriginal children had dry perforations, 15% Aboriginal children had AOM, 19% Aboriginal children had AOM with perforation, while 58% of Aboriginal children with a suppurative OM diagnosis had chronic suppurative OM. Respiratory bacterial counts were high in these groups, and did not differ significantly between groups (p = 0.39).

**Figure 1:**
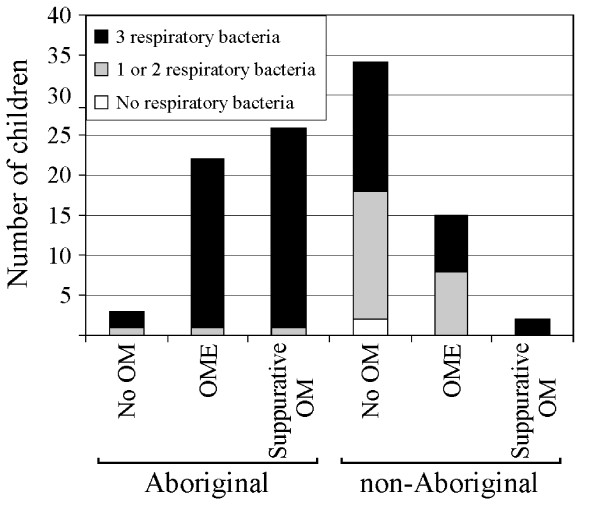
Respiratory bacterial carriage in Aboriginal and non-Aboriginal children determined by RTQ-PCR.

All swabs tested from Aboriginal children and 50/52 swabs from non-Aboriginal children were positive for at least one of the respiratory pathogens, *S. pneumoniae, H. influenzae *or *M. catarrhalis *(Figure 1). The proportion of swabs positive for all three pathogens was significantly higher for Aboriginal children compared with non-Aboriginal children (48/51 versus 25/52, *P *= 0.0001).

### Density of nasal bacteria measured by RTQ-PCR

**Figure 2:**
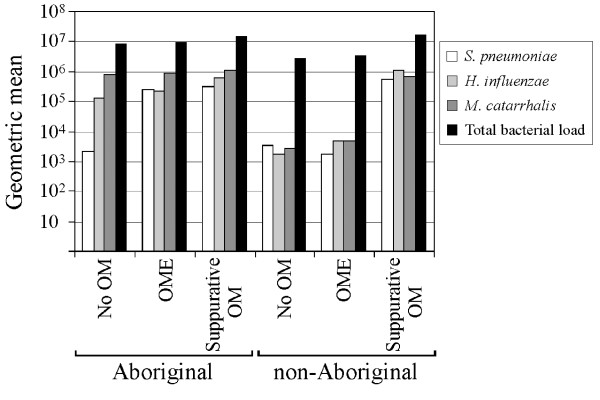
Level of *S. pneumoniae*, *H. influenzae*, *M. catarrhalis *and total bacterial load by ear state in Aboriginal and non-Aboriginal children as determined by RTQ-PCR.

Species-specific individual bacterial RTQ-PCR counts ranged from zero to 3.9 × 10^7 ^cells (ml of sample)^-1^. The geometric mean counts for Aboriginal children were generally higher than for non-Aboriginal children (Figure 2).

For Aboriginal children, *H. influenzae *geometric mean counts were similar, regardless of ear state and increased by only 0.5-log for suppurative OM. *M. catarrhalis *geometric mean RTQ-PCR counts were considerably higher than other species, with a smaller margin of increase between ear states. The *S. pneumoniae *geometric mean RTQ-PCR count for no OM was considerably lower than other species and increased by 2-logs for OME and for suppurative OM.

In non-Aboriginal children, geometric mean respiratory bacterial counts for all species were comparable for no OM and OME; a considerable increase (2 to 2.5-logs) for all species was detected in suppurative OM (2 children).

For children with no OM or OME, Aboriginal children had higher geometric mean total bacterial loads than non-Aboriginal children (Figure 2). For Aboriginal and non-Aboriginal children, total bacterial counts were comparable with suppurative OM, and higher than no OM or OME (Figure 2).

### The ratio of respiratory pathogen load to total bacterial load

**Figure 3:**
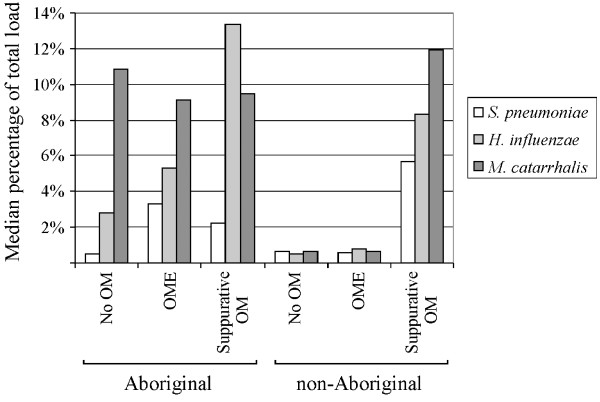
*S. pneumoniae*, *H. influenzae *and *M. catarrhalis *as a percentage of total load by ear state in Aboriginal and non-Aboriginal children as determined by RTQ-PCR.

The ratio of respiratory pathogen load to total bacterial load was estimated as a percentage for each swab (Figure 3). Total bacterial load varied between 7 × 10^4 ^and 2 × 10^8 ^cells (ml of sample)^-1^. *S. pneumoniae, H. influenzae *or *M. catarrhalis *counts as a proportion of total bacterial load reached 46%, 78%, and 65%, respectively.

In the Aboriginal children, *S. pneumoniae *was a small proportion of total bacterial load for no OM (<1%), increasing only slightly for OME (3%) and suppurative OM (2%). The *H. influenzae *ratio was higher than *S. pneumoniae*, increasing progressively through the ear states from 3% for no OM to 5% for OME to 13% for suppurative OM. *M. catarrhalis *proportions remained at approximately 10%. In the non-Aboriginal children, each of the respiratory pathogens was a low proportion of the total load for no OM and OME (<1%), but increased substantially for suppurative OM (6% for *S. pneumoniae*, 8% for *H. influenzae *and 12% for *M. catarrhalis*).

### Comparison of Aboriginal and non-Aboriginal groups with OME

For children with OME (22 Aboriginal and 16 non-Aboriginal), *S. pneumoniae *and *M. catarrhalis *nasal load (Figure 2) and proportion of total load (Figure 3) were significantly increased in the Aboriginal group (Mann-Whitney U test, *P *= 0.0001 – 0.03). Comparison of *H. influenzae *load between Aboriginal and non-Aboriginal children with OME did not reach significance due to the large variability in the *H. influenzae *data for the OME group, particularly for the non-Aboriginal children. The interquartile range of *H. influenzae *as a proportion of total load in non-Aboriginal and Aboriginal children was 26.3 and 15.5, respectively, compared with 2.1 and 9.5 for *S. pneumoniae*, and 2.2 and 12.2 for *M. catarrhalis*.

### Association between nasal bacterial load and ear state

**Figure 4:**
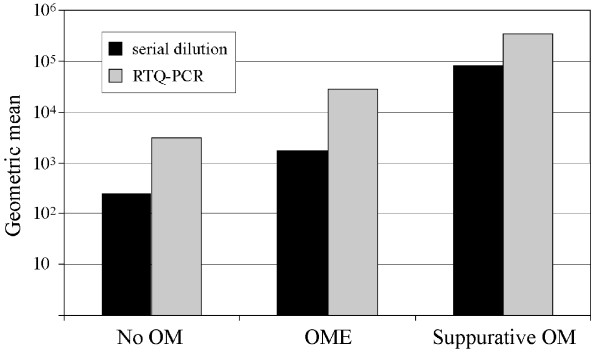
Relationship between the level of *S. pneumoniae *and ear state.

The Kruskal-Wallis non-parametric comparison of means test was used to measure the association between bacterial load and ear state severity for the combined populations. As only three children in the Aboriginal group had a No OM ear diagnosis, and two children in the non-Aboriginal group had a Suppurative OM diagnosis, separate analyses within groups could not be undertaken. *S. pneumoniae *load, measured by serial dilution colony counts or RTQ-PCR, was positively associated with ear state (*P *= 0.0001–0.0016; Figure 4) as were *H. influenzae *and *M. catarrhalis *(data not shown). Total nasal bacterial load measured by RTQ-PCR was also positively associated with ear state (*P *= 0.0001), as was the ratio of respiratory pathogens to total bacterial load (*P *= 0.0032).

**Figure 5:**
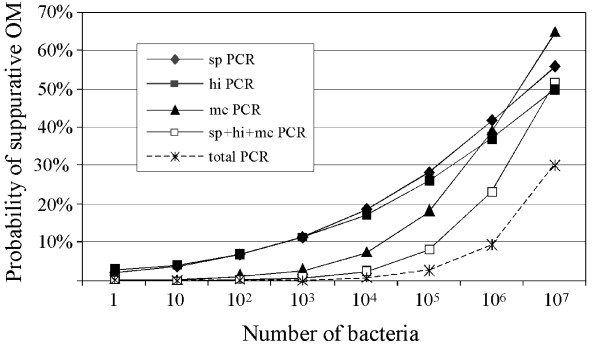
Predicted probability of suppurative OM with bacterial load based on the logistic regression model of data acquired by RTQ-PCR.

In order to assess the utility of bacterial load as a predictor of outcome, the probability of a suppurative OM diagnosis across the range of bacterial counts was determined. The analysis showed that the probability of suppurative OM increases with the density of *S. pneumoniae, H. influenzae*, or *M. catarrhalis*, combined pathogen load, and total bacterial load as determined by RTQ-PCR (Figure 5). Compared with *M. catarrhalis*, both *S. pneumoniae *and *H. influenzae *were associated with a higher probability of suppurative OM at lower counts. Both the combined respiratory pathogen load and total bacterial load were only associated with a greater probability of suppurative OM at high counts (Figure 5).

For swabs that were culture-negative and RTQ-PCR-positive for *S. pneumoniae *(18 of 103 swabs) and *H. influenzae *(24 of 103 swabs), there was a statistically significant association with no OM or OME (*P *= 0.0001). For similar samples of *M. catarrhalis *(6 of 103 swabs) there was no association with ear state.

A further comparison was made with routine semi-quantitative assessment of bacterial load established in our laboratory. Despite the lower precision of this simple method, a significant association was demonstrated for all bacteria tested and presence and severity of ear disease (Chi^2 ^exact; *S. pneumoniae*, *P *= 0.003; *H. influenzae*, *P *= 0.012; *M. catarrhalis*, *P *= 0.013).

### Selection of a threshold load for predicting OM

**Figure 6:**
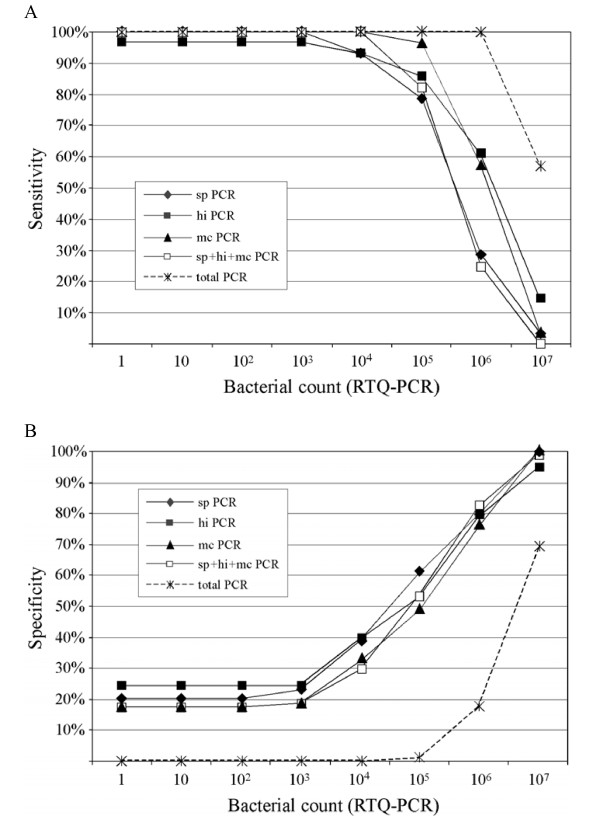
Sensitivity (A) and specificity (B) for predicting suppurative OM with bacterial load based on data acquired by RTQ-PCR.

Using sensitivity and specificity measures, threshold bacterial load values that could identify children most likely to have suppurative OM from those less likely to be affected were estimated. For example, the sensitivity of an RTQ-PCR estimate of 10^5 ^cells (ml of sample)^-1 ^for predicting suppurative OM was 79% to 96% for individual pathogenic species, and 82% for combined respiratory pathogens (Figure 6a). Specificity ranged from 49% to 61% for individual, and 52% for combined respiratory pathogens (Figure 6b). These analyses showed that high nasal respiratory pathogen counts occurred in children without OM, but that a low count was unlikely in a child with suppurative OM. Analyses of serial dilution colony count data and RTQ-PCR data for predicting suppurative OM or any OM showed similar results.

## Discussion

### Positive correlation between nasal bacterial load and ear state

In this study, positive correlations between nasal bacterial load and ear state (no OM, OME, or suppurative OM) were observed. This relationship held for single and combined bacterial respiratory pathogens (*S. pneumoniae, H. influenzae*, or *M. catarrhalis*), total bacterial load, and the proportion of respiratory pathogens to total bacterial load.

### Nasal bacterial load as a diagnostic marker

Nasal bacterial load of respiratory pathogens was a highly sensitive measure of suppurative OM, but had low specificity, and is therefore not a reliable diagnostic tool. Nevertheless, the high sensitivity supports the view that a low bacterial load makes the presence of suppurative OM unlikely.

### Comparison of nasal bacterial load in Aboriginal and non-Aboriginal children

Comparison of Aboriginal and non-Aboriginal children with OME demonstrated significantly higher loads of *S. pneumoniae *and *M. catarrhalis *in the Aboriginal group. Furthermore, the proportion of swabs positive for all three pathogens was significantly higher for Aboriginal children compared with non-Aboriginal children. We believe that the high-density carriage of multiple respiratory bacteria in Aboriginal children is a result of high rates of cross-infection driven by overcrowding and poor health infrastructure, long carriage times, and immune suppression resulting from inadequate nutrition and early age of first infection [1,8,9]. Furthermore, we hypothesise that high-density carriage of *S. pneumoniae, H. influenzae*, and *M. catarrhalis *predicts persistence of OME, and progression to suppurative OM.

### Superior detection of bacterial respiratory pathogens by RTQ-PCR

RTQ-PCR proved to be the more sensitive method for quantifying *S. pneumoniae, H. influenzae*, and *M. catarrhalis *in nasal swabs. For *S. pneumoniae*, this may be due to diplococci or chains of diplococci counted as a single colony on culture plates, compared to more precise enumeration by PCR [19]. Bacterial death in storage may be a contributing factor. However, we have detected only a minor change in viable counts over 6 years of storage. Another explanation may be the detection of released DNA in the nose. However, evidence suggests that DNA is cleared efficiently from mucosal surfaces through a combination of bacterial lysis and immune clearance mechanisms, since bacterial DNA does not survive in an amplifiable form for more than three days in the presence of a middle ear effusion[20]. Antibiotic use within 4 weeks of swab collection was documented for only 3 children and does not explain this phenomenon. Thus the most likely explanation for the culture-negative, RTQ-PCR-positive events relates to the suppressed metabolic state which enables many different bacterial species to survive in a non-cultivable state [21,22]. Several hypotheses exist to explain this phenomenon[23].

### Clinical relevance of non-cultivable bacteria

Non-cultivable forms of *S. pneumoniae *and *H. influenzae *in middle ear effusions elicit an immune response and may have a crucial role in the pathogenesis of OME [24]. In our study, the presence of these bacteria in nasal swabs was associated with no OM or non-acute OM (OME). Others have demonstrated that whilst culture-negative, PCR-positive pneumococcal acute OM does occur, this is clinically less severe than culture-positive pneumococcal acute OM[19].

Of particular interest is whether non-cultivable bacteria can recrudesce or have a direct pathogenic role in persistence of effusions, or in recurrent acute OM. With an increasing trend towards the use of PCR-based diagnostics, it is important to clarify the role of non-cultivable organisms in disease.

## Conclusion

This study demonstrated a significant association between nasal bacterial load and the presence and severity of current ear disease in individuals. These associations persisted whether bacterial load was measured by a semi-quantitative culture method, serial colony counts, or by RTQ-PCR. Detection of non-cultivable bacteria by RTQ-PCR may have implications for the pathogenesis of OM including persistence of infection despite aggressive therapies.

Swabs with higher bacterial loads were more likely to have been obtained from Aboriginal children with suppurative OM. However, bacterial load measurements were not reliable enough in distinguishing children with suppurative OM to be of diagnostic use. Notwithstanding the low specificity of bacterial load as a measure of ear state, the high sensitivity supports the view that a low bacterial load makes the presence of suppurative OM unlikely. This observation also suggests that reductions in bacterial load may be essential before dramatic reductions in suppurative OM can be achieved for Aboriginal children living in remote communities of Australia.

## Authors' contributions

HS-V participated in the design of the study, carried out the microbiological analysis, and drafted the manuscript.

RB developed and carried out the molecular analysis.

MN developed and carried out the molecular analysis, and participated in the design of the study.

NAJ and NH coordinated the development of the molecular techniques, and participated in the design of the study.

SH performed the statistical analysis.

AJL and PSM conceived the study, were chief investigators of the original studies from which the swabs were derived, and participated in the design of the study.

## Pre-publication history

The pre-publication history for this paper can be accessed here:


